# Requirement of Pdgfrα^+^ cells for calvarial bone repair

**DOI:** 10.1093/stcltm/szae041

**Published:** 2024-07-10

**Authors:** Xin Xing, Zhao Li, Jiajia Xu, Austin Z Chen, Mary Archer, Yiyun Wang, Mingxin Xu, Ziyi Wang, Manyu Zhu, Qizhi Qin, Neelima Thottappillil, Myles Zhou, Aaron W James

**Affiliations:** Department of Pathology, Johns Hopkins University, Baltimore, MD 21205, United States; Department of Pathology, Johns Hopkins University, Baltimore, MD 21205, United States; Department of Pathology, Johns Hopkins University, Baltimore, MD 21205, United States; Department of Pathology, Johns Hopkins University, Baltimore, MD 21205, United States; Department of Pathology, Johns Hopkins University, Baltimore, MD 21205, United States; Department of Pathology, Johns Hopkins University, Baltimore, MD 21205, United States; Department of Pathology, Johns Hopkins University, Baltimore, MD 21205, United States; Department of Pathology, Johns Hopkins University, Baltimore, MD 21205, United States; Department of Pathology, Johns Hopkins University, Baltimore, MD 21205, United States; Department of Pathology, Johns Hopkins University, Baltimore, MD 21205, United States; Department of Pathology, Johns Hopkins University, Baltimore, MD 21205, United States; Department of Pathology, Johns Hopkins University, Baltimore, MD 21205, United States; Department of Pathology, Johns Hopkins University, Baltimore, MD 21205, United States

**Keywords:** platelet-derived growth factor receptor, mesenchymal cells, skeletal cells, single-cell RNA sequencing, calvarial bone, bone repair, bone healing

## Abstract

Platelet-derived growth factor receptor α (PDGFRα) is often considered as a general marker of mesenchymal cells and fibroblasts, but also shows expression in a portion of osteoprogenitor cells. Within the skeleton, Pdgfrα^+^ mesenchymal cells have been identified in bone marrow and periosteum of long bones, where they play a crucial role in participating in fracture repair. A similar examination of Pdgfrα^+^ cells in calvarial bone healing has not been examined. Here, we utilize Pdgfrα-CreER^TM^;mT/mG reporter animals to examine the contribution of Pdgfrα^+^ mesenchymal cells to calvarial bone repair through histology and single-cell RNA sequencing (scRNA-Seq). Results showed that Pdgfrα^+^ mesenchymal cells are present in several cell clusters by scRNA-Seq, and by histology a dramatic increase in Pdgfrα^+^ cells populated the defect site at early timepoints to give rise to healed bone tissue overtime. Notably, diphtheria toxin-mediated ablation of Pdgfrα reporter^+^ cells resulted in significantly impaired calvarial bone healing. Our findings suggest that Pdgfrα-expressing cells within the calvarial niche play a critical role in the process of calvarial bone repair.

## Graphical Abstract

**Graphical Abstract:**
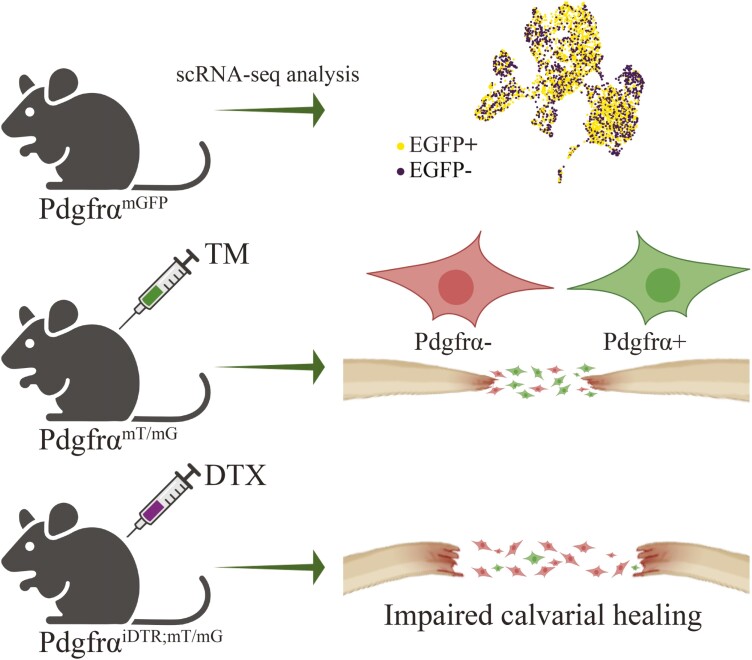

**Significance statement**

The present work has identified that high Pdgfrα expression serves as a marker for distinct subpopulations of mesenchymal progenitor cells within the calvaria niche. Pdgfrα^+^ cells located in the calvaria are associated with intramembranous ossification, showing a heightened tendency to participate in proliferation and osteogenesis. Depletion of Pdgfrα^+^ cells result in impairments in calvarial bone repair. The present findings emphasize the significance of the presence of Pdgfrα^+^ cells in maintaining bone homeostasis and facilitating repair within the calvaria.

## Introduction

Skeletal mesenchymal/progenitor cells are present within the bone microenvironment and participate in skeletal development, homeostasis, and repair.^1^ In mice, various subpopulations of skeletal mesenchymal/progenitor cells have been identified within the bone marrow stroma, marked by lineage tracing or specific markers such as Osterix (Osx),^2^ Myxovirus resistance-1 (Mx1),^3^ Gremlin 1 (Grem1),^4^ and GLI family zinc finger 1 (Gli1).^5^ Recent progress has expanded our knowledge to include mesenchymal/progenitor cells in skeletal tissues beyond the bone marrow, such as cranial sutures and the periosteum, identified by markers such as Gli1,^6^ Axin2,^7^ and paired-related homeobox 1 (Prx1).^8^ Moreover, markers such as Prx1,^9^ α-smooth muscle actin (αSMA),^10^ Cathepsin K (Ctsk),^11^ and SRY-box transcription factor 9 (Sox9)^12^ have been described for long bone periosteal progenitor cells contributing to bone repair.^13,14^ These findings indicate that endogenous skeletal progenitor cells may exist in multiple tissue locations within adult bones.

Platelet-derived growth factor (PDGF) is a potent mitogen and chemoattractant for many cell types, including skeletal cells, facilitating vital processes for bone repair, including cell migration, proliferation, and angiogenesis.^15-18^ PDGF mediates its signal through high-affinity transmembrane receptors endowed with intrinsic tyrosine kinase activity. Among these receptors, platelet-derived growth factor receptor α (PDGFRα) is often considered a general marker for fibroblasts and mesenchymal cells, and a useful identifier of progenitor cell populations across multiple mesodermal tissues, including muscle and bone marrow.^19-21^ Several mouse reporter systems for PDGFRα have been devised,^22-25^ including inducible reporter systems.^26,27^ Notably, our laboratory has previously used an inducible Pdgfrα reporter animal to identify perivascular progenitor cell subpopulations within adipose tissue.^28^ Indeed, using the same reporter system we found that Pdgfrα reporter activity identifies functionally relevant fractions of periosteum within mouse long bones.^29^

In the context of bone healing, 2 distinct and well-delineated ossification mechanisms come into play: intramembranous and endochondral ossification.^30^ Endochondral ossification represents an indirect approach to bone formation, commonly observed during the repair of long bone fractures. In this process, mesenchymal progenitor cells initially form a cartilage template (anlage), which eventually transforms into bone tissue. On the other hand, craniofacial bones, such as the calvarium, exhibit a different pattern. Comprising flat bones, their healing process predominantly follows intramembranous ossification, where bone develops directly from mesenchymal progenitor cells, bypassing the intermediate step of cartilage formation.^31^ It is worth noting that tissue-resident skeletal mesenchymal/progenitor cells comprise a diverse and heterogeneous population located in various regions. A previous study suggested that the deficiency of Pdgfrα is associated with alterations in the morphogenesis of both frontal and parietal bones during embryonic calvarial development.^32^ The question of whether Pdgfrα^+^ mesenchymal/progenitor cells are present in the adult calvarial cellular niche and play a role in calvarial bone healing remains essentially unexplored.

Here, we used single-cell RNA sequencing (scRNA-seq) analysis, unveiling a significant enrichment and diversity of Pdgfrα expressing mesenchymal cells within mouse full thickness calvarial defects. Subsequently, we used tamoxifen-inducible Pdgfrα reporter animals to identify the involvement of Pdgfrα^+^ cells in calvarial defect bone healing. Using cell depletion studies, we found that ablation of Pdgfrα-expressing cells led to significantly impaired calvarial bone healing. These findings underscore the critical role of Pdgfrα^+^ cells in calvarial bone repair and contribute to the burgeoning understanding of craniofacial bone healing.

## Materials and methods

### Mice

All animal experiments strictly adhered to approved protocols (MO19M266, MO19M366, and MO22M368) of the Animal Care and Use Committee (ACUC) at Johns Hopkins University (JHU). The Pdgfrα-CreER animals used in this study were generously provided by the Dwight Bergles laboratory^33^ and are commercially available through the Jackson Laboratory (Stock No. 018280). The generation of Pdgfrα^mT/mG^ mice involved crossing Pdgfrα-CreER mice with mT/mG mice (JAX Stock No. 007576). Pdgfrα^iDTR;mT/mG^ mice were created by crossing Pdgfrα^mT/mG^ mice with iDTR mice (JAX Stock No. 007900). Male mice were used for all experiments. Tamoxifen (TM; Sigma-Aldrich) and diphtheria toxin (DTX; Sigma-Aldrich) were administered intraperitoneally in accordance with established protocols (TM: 150 mg·kg^−1^ per day for 5 days; DTX: 45.7 μg·kg^−1^ per day for 3 days).^29,34^ TM was dissolved in sunflower seed oil (Sigma-Aldrich).

### Single-cell RNA sequencing

scRNA-seq data were obtained from our previously published expression data of mouse calvarial cells, (GEO accession code GSE179891).^35^ In this study, male Pdgfrα^mT/mG^ animals, aged 2 months, received tamoxifen treatment 14 days prior to the creation of a circular frontal bone defect with a 1.8-mm diameter. Analysis was conducted 7 days postoperatively. Raw sequencing reads in FASTQ format were aligned, filtered, barcode-assigned and unique molecular identifier (UMI)-counted using the Cell Ranger v7.1.0 software suite (10× Genomics) with a custom genome reference based on the mouse (mm10)-2020-A genome supplemented with sequences of the EGFP transgenes used in our experiments. Doublets were removed using Scrublet with an expected doublet rate of 10% and automatically set threshold at a doublet score of 0.77. Downstream analyses were performed using Scanpy v1.9.4. In pre-processing, cells expressing fewer than 200 genes or with over 15% of UMIs mapping to mitochondrial genes were excluded from further analysis. Filtered data containing 3500 highly variable genes (HVGs) were normalized and log-transformed using SCANPY workflows to account for technical noise. Unsupervised clustering was performed by Leiden cluster module in SCANPY. Differentially expressed genes (DEGs) between clusters were identified using Wilcoxon rank-sum tests. Pseudotime trajectories for osteogenesis and fibrogenesis were generated using Monocle 2 v2.24.0 in R. We identified and ranked the top 500 differentially expressed genes (DEGs) across all clusters. These DEGs were then used to reduce the expression profile to 2 dimensions via the DDRTree algorithm, allowing for the sorting of cells and the creation of a trajectory map. Pseudotime values were incorporated as metadata in the Scanpy object and analyzed using the Scanpy package.

### Mouse calvarial defect model

The procedure for creating calvarial defects followed established protocols.^36^ Prior to the surgery, mice received an intraperitoneal injection of an analgesic (1 mg/kg buprenorphine ER) for pain management. The process involved shaving the hair over the calvaria and preparing the skin in a sterile manner using alternating betadine and alcohol scrubs. Throughout the experiment, mice were maintained under deep anesthesia via isoflurane gas inhalation (2% isoflurane in 22% oxygen/78% nitrogen inhalation). A sagittal incision, measuring 1 cm in length, was meticulously made along the midline of the skull to expose the frontal bone. Employing a microsurgical drill and a trephine drill bit, a 1.8 mm diameter full-thickness circular defect was carefully created on the frontal bone, with special attention given to preserving neighboring sutures and underlying tissues. The calvarial defect sites were irrigated with normal saline. Subsequently, the incision was sutured, and the animal was closely monitored according to established postoperative protocols. Skulls were collected at various time points, with assessments extending up to 28 days following the injury.

### Radiographic analyses

Skulls underwent a thorough dissection procedure to carefully remove the surrounding skin and brain tissue. Following this dissection, they were subjected to evaluation using a high-resolution micro-CT imaging system (SkyScan1275, Bruker, Kontich, Belgium). During scanning, images were acquired at an image resolution of 10 μm, with the following parameters: X-ray voltage set at 65 kVp, anode current of 153 mA, exposure time ranging from 65 to 218 ms, frame averaging of 5, and a rotation step of 0.3 °. Three-dimensional images were reconstructed from the 2-dimensional X-ray projections using the Feldkamp algorithm, employing the commercial software package NRecon (version 2.0.4.0, SkyScan). For the subsequent morphometric analyses of the images, we used the CTVox and CTAn software (version 1.13, SkyScan). In the assessment of calvarial defects, a cylindrical volume of interest was centered around each defect site. This volume had a diameter of 1.8 mm and a height of 1 mm. A threshold value of 80-255 was applied for segmentation within this segmented volume. From this segmented volume, we derived measurements for bone volume (BV) and tissue volume (TV). Additionally, we determined the bone fractional area (BFA) and residual defect diameter. This was accomplished by creating a top-down 3D rendering of the calvarial defect using CTVox and subsequently measuring it using ImageJ software (version 1.8.0; National Institutes of Health, Bethesda, MD, USA). To evaluate bone healing, a semiquantitative score ranging from 0 to 4 was assigned by 3 blinded observers. This scoring system was based on previously published grading scales for calvarial defect healing^37^ and followed the criteria below: 0 indicated no bone formation, 1 indicated a few dispersed bony spicules within the defect, 2 indicated bony bridging limited to the defect borders, 3 indicated partial bony bridging along the defect length, and 4 indicated complete bony bridging spanning the entire defect at its longest point.

### Histologic and immunohistochemical analyses

Tissues were fixed in 4% paraformaldehyde (PFA) for 12 hours, followed by decalcification in 14% EDTA for 14 days at 4 °C on a shaker, and subsequent embedding in optimal cutting temperature compound (OCT; Sakura, Torrance, CA). Coronal sections of the calvaria, 20 μm in thickness, were obtained using cryosectioning. Hematoxylin and eosin (H&E) staining was performed according to routine procedure. Alkaline phosphatase (ALP) staining was carried out in accordance with the manufacturer’s instructions. For immunofluorescent staining, sections were washed in PBS and permeabilized with 0.5% Triton-X for 30 minutes. Subsequently, sections were blocked using SuperBlock blocking buffer (Thermo Fisher Scientific) and incubated with primary antibodies overnight at 4 °C in a humidified chamber (Table 1 for a summary of the antibodies used). The following day, the slides were washed in PBS and incubated with the appropriate secondary antibodies for 1 hour at RT. Finally, the sections were mounted with DAPI mounting solution (Vectashield H-2000, Vector Laboratories, Burlingame, CA). Digital images of these sections were captured using either upright fluorescent microscopy (Leica DM6, Leica Microsystems Inc., Buffalo Grove, IL) or confocal microscopy (Zeiss LSM900 FCS, Carl Zeiss Microscopy GmbH, Jena, Germany).

**Table 1.** Antibodies used.

Reagent	Resource	Identifier
Rabbit polyclonal Anti-Ki67	Abcam	Cat# ab15580
Rabbit polyclonal Anti-Osteocalcin	Abcam	Cat# ab93876
Rabbit polyclonal Anti-Runx2	Abcam	Cat# ab192256
Mouse monoclonal Anti-Collagen I	Abcam	Cat# ab88147
Goat Anti-Rabbit IgG H&L (Alexa Fluor 647)	Abcam	Cat# ab150079
Goat Anti-Mouse IgG H&L (Alexa Fluor 647)	Abcam	Cat# ab150119

### Statistical analyses

The data are presented as the mean ± 1 standard deviation (SD). A Shapiro-Wilk test for normality was performed for all datasets. Statistical analysis consisted of a 2-sample *t*-test, and one-way analysis of variance (ANOVA) with post hoc Dunn’s multiple comparisons test was conducted using GraphPad Prism 8. The number of animals used in the in vivo experiments is shown in the figure legends. Sample size calculations were performed for cell depletion experiments presented in Figures 6 based on an anticipated effect size of 2.58, using our previously published data in adult mice.^29^ For this scenario, with 6 samples per group, a 2-sample *t* test would provide 80% power to detect effect sizes of at least 2.0 assuming a 2-sided 0.05 level of significance.

**Figure 1.**
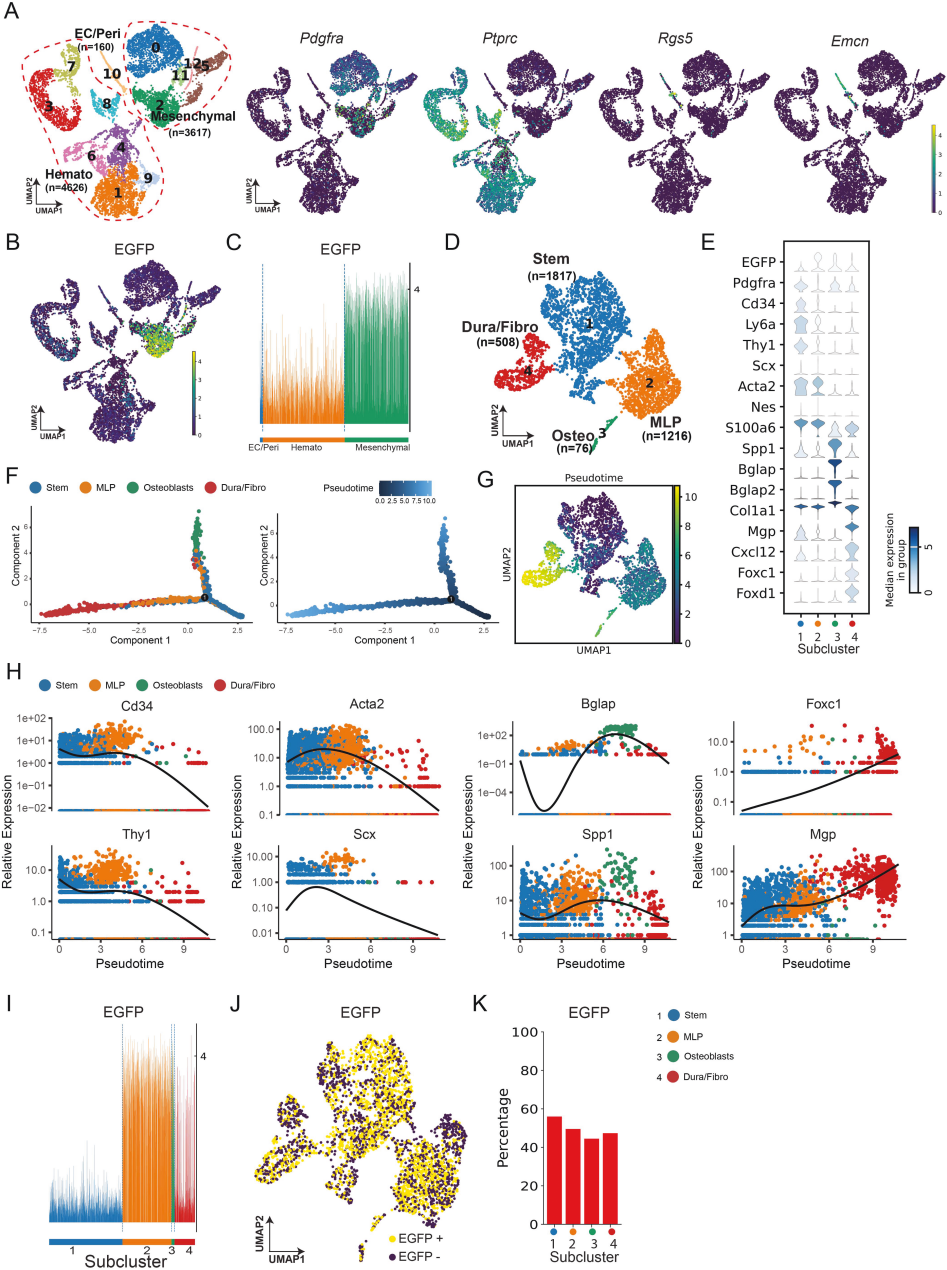
Single-cell analysis of mouse calvarial defect identifies mesenchymal cells participating in repair. Dataset was collected from 2-month-old male Pdgfrα^mT/mG^ mice with a 1.8-mm circular calvarial defect, analyzed 7 days postsurgery. (A) Uniform manifold approximation and projection (UMAP) plot of total 8403 cells isolated from the calvarial defect site of Pdgfrα^mT/mG^ mice (*n* = 4 mice), with UMAP visualization of selective marker gene expression for mesenchymal, EC/Peri, and hemato cell clusters. Cell numbers are listed in parenthesis next to cluster names. Color intensity indicates average expression level. (B) Feature plot of EGFP expression for mesenchymal, EC/Peri, and hemato cell clusters. (C) Trackplot showing the distribution of EGFP expression across all the 3 main clusters. (D) UMAP plot of only mesenchymal cells (total 3617 cells, clusters 0, 2, 5, 11, and 12 in A). (E) Stacked violin plot of marker gene expression for each cell cluster. (F) Differentiation trajectory of mesenchymal cells reconstructed by monocle2. Each dot indicates a single cell, with color coding showing cell clusters. (G) UMAP plot of pseudotime value. (H) The expression pattern of selected marker genes along the pseudotime. (I) Distribution of EGFP expression across the 4 subclusters by trackplot. (J) Feature plot showing EGFP expression pattern across 4 mesenchymal subclusters. (K) Bar plot showing the percentage of EGFP-positive cells across different subclusters. Abbreviations: EC, endothelial cell; Peri, pericyte; hemato, hematopoietic; Osteo, osteoblast; MLP, multilineage progenitor; Dura/fibro, dura/fibroblast.

**Figure 2.**
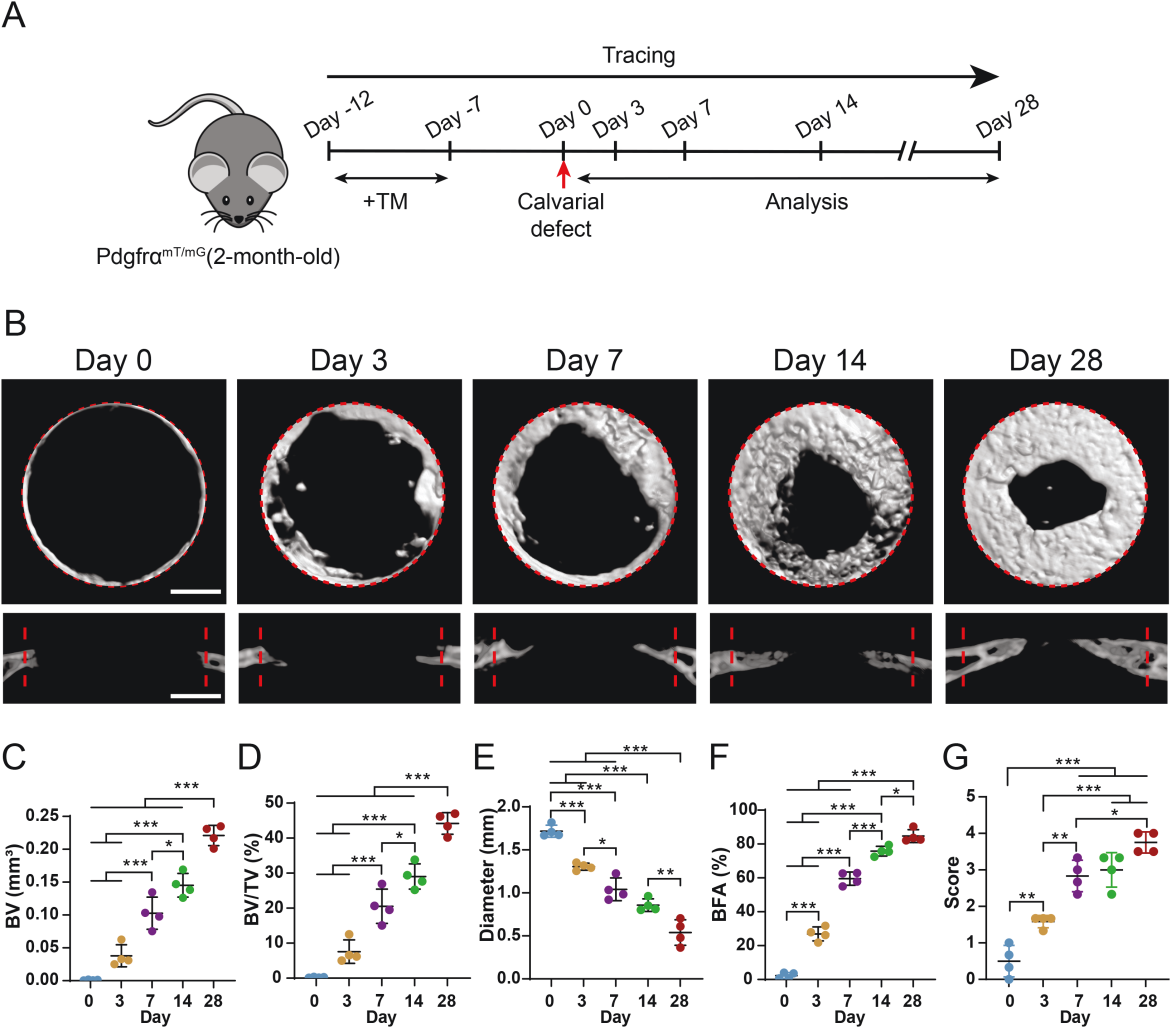
Calvarial defect healing patterns over time in Pdgfrα reporter animals as assessed by micro-CT. (A) Schematic of the experiment: Pdgfrα^mT/mG^ animals (male, aged 2 months) were administered tamoxifen (TM). Bone healing and reporter activity was examined 0, 3, 7, 14, and 28 days after injury. (B) Micro-CT reconstructions of the defect site in a top-down view (above) and coronal cross-sectional images (below) in Pdgfrα^mT/mG^ animals at different postinjury time points. Margins of original defect are indicated by dashed lines. Micro-CT quantification of bone parameters at different postinjury time points, including (C) bone volume (BV), (D) bone volume/tissue volume (BV/TV), (E) residual defect diameter, (F) bone formation area (BFA), and (G) bone healing score (score). Each dot represents a single animal. Scale bars, 500 μm. Data are represented as means ± 1 SD; *n* = 4 mice. *P < .05, **P < .01, and ***P < .001 as assessed using one-way ANOVA test.

**Figure 3.**
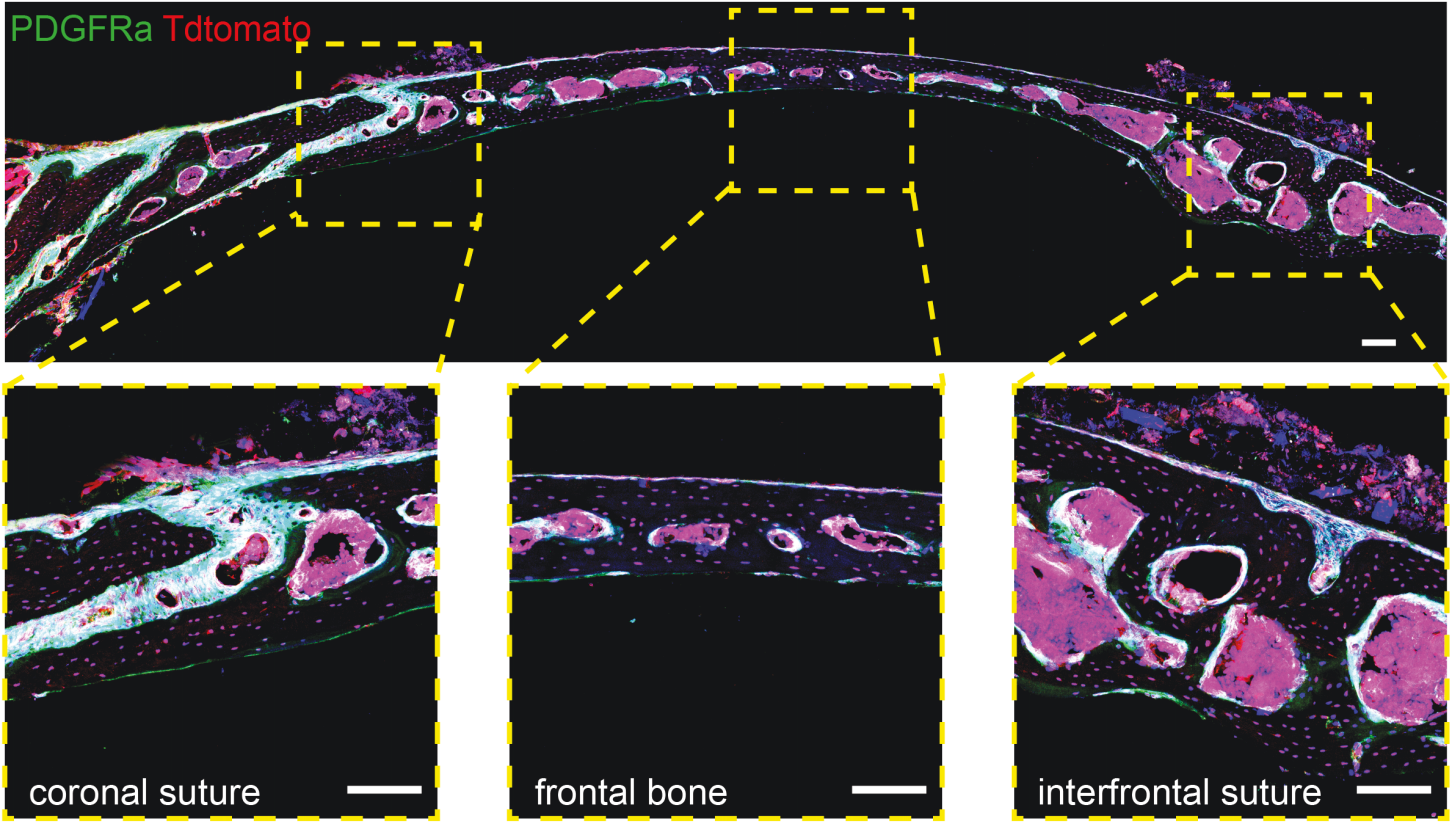
PDGFRα reporter activity in the uninjured mouse calvaria. Pdgfrα^mT/mG^ animals (male, 2 months old) were administered tamoxifen (TM) for 5 consecutive days, followed by a 7 days washout period. Reporter activity was assessed in the uninjured calvaria. Scale bar: 100 μm.

**Figure 4.**
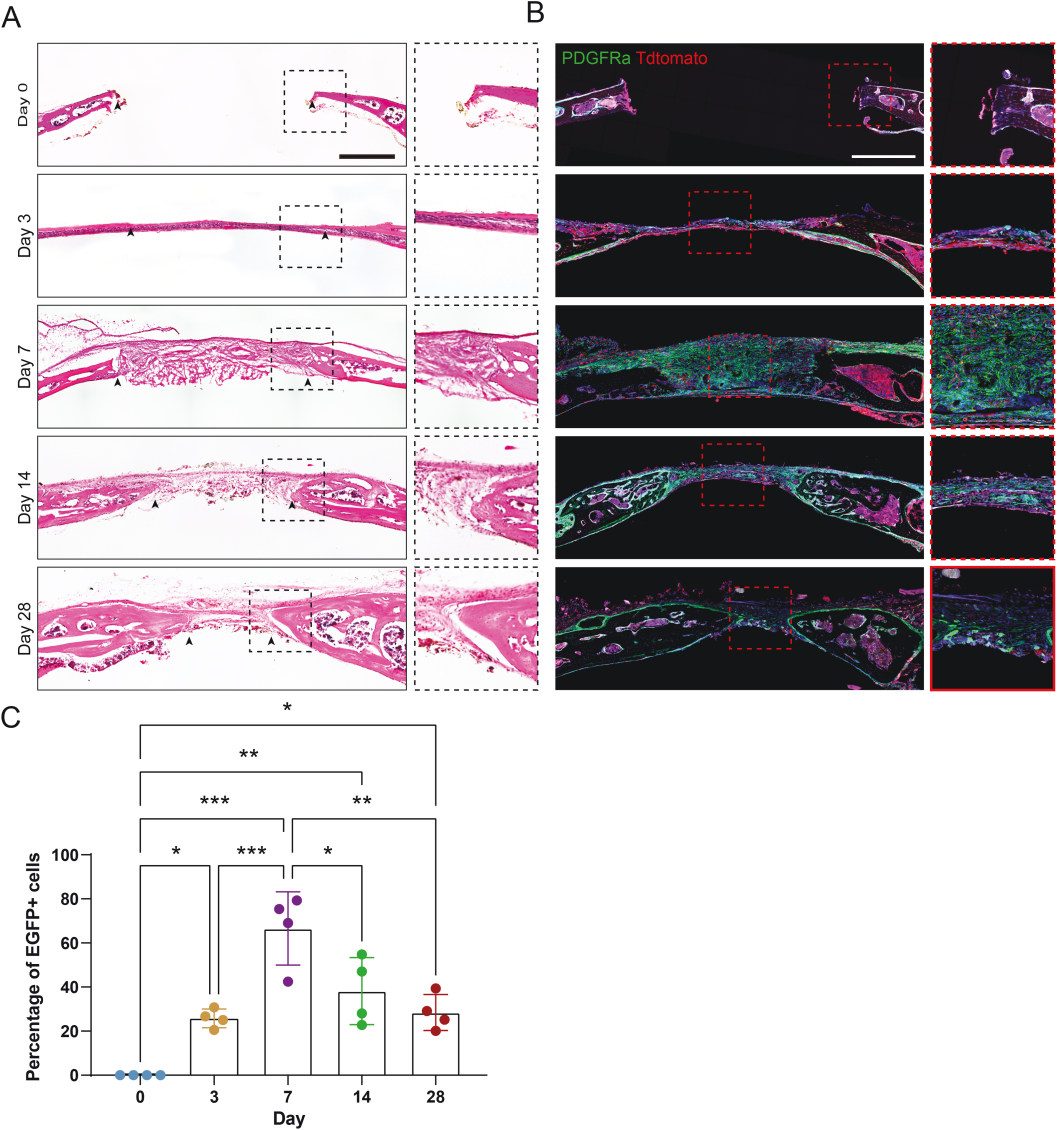
Dynamic changes in Pdgfrα reporter across time over calvarial defect healing. Pdgfrα^mT/mG^ mice (male, 2 months) received tamoxifen (TM), and reporter activity was evaluated postinjury. (A) Haematoxylin and eosin (H&E) staining of coronal cross section of the healing defect site at different time points after injury (0, 3, 7, 14, and 28 days). Black arrowheads indicate healing bone edges. (B) Reporter activity of the Pdgfrα reporter in 0, 3, 7, 14, and 28 days postinjury. (C) The percentages of EGFP-positive cells were calculated based on EGFP marker-positive cell numbers per DAPI-positive cell numbers. Scale bar: 500 μm. Data are represented as mean ± 1 SD; *n* = 4 mice. Each dot represents a single animal. *P < .05, **P < .01, and ***P < .001 as assessed using one-way ANOVA test.

**Figure 5.**
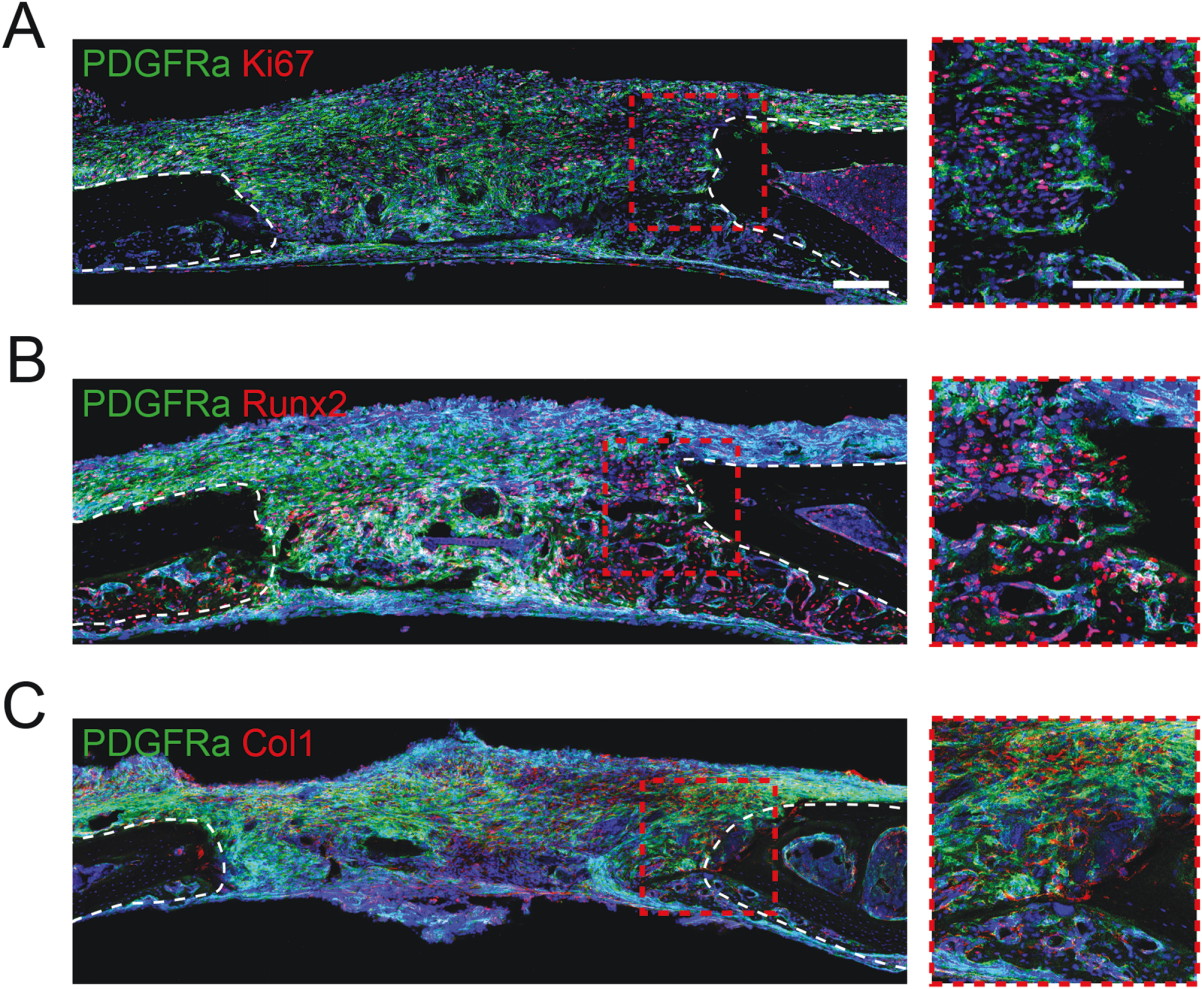
Immunohistochemical analysis of Pdgfrα^mT/mG^ injured calvarial defects. (A) Representative immunohistochemical images of Ki67 expression at 7 days after injury. (B) Representative immunohistochemical staining image of Runt-related transcription factor 2 (Runx2) at 7 days after injury. (C) Representative immunohistochemical staining images of collagen type 1 (Col1) expression 7 days after injury. Scale bar: 200μm

## Results

### scRNA-seq revealed that Pdgfrα is highly expressed in the mesenchymal cell cluster of calvarial defect

First, we conducted a re-analysis of our previously generated calvarial single-cell sequencing dataset, that included a total of 8403 cells isolated from the calvarial defect site of Pdgfrα^mT/mG^ mice at 7 days after injury. The specificity of Cre recombination has been validated, where enhanced green fluorescent protein (EGFP) expression was confined to Pdgfrα-expressing cells.^35^ Through unsupervised clustering analysis, we identified the presence of 13 distinct cell subclusters including 5 groups of mesenchymal cells which expressed *Pdgfra*,^19^ and 7 groups of hematopoietic cells expressing protein tyrosine phosphatase receptor type C (*Ptprc*),^38^ and 1 group of endothelial cells/pericytes by the expression of previously defined markers regulator of G-protein signaling 5 (*Rgs5*)^39^ and endomucin (*Emcn*)^40^ (Figure 1A). The markers used to define each of the 13 distinct cell subclusters are displayed by dot plot (Supplementary Figure S1). The feature plot depicting the expression pattern of the EGFP gene across all cell subpopulations, showed a similar expression pattern as *Pdgfrα*. It is noticeable that EGFP is highly expressed in mesenchymal lineage cells, particularly within cluster 2 (Figure 1B). The trackplot further confirmed the high EGFP expression at single-cell resolution in mesenchymal cells, in comparison to hematopoietic and endothelial cells/pericytes (Figure 1C). By subclustering the 3617 mesenchymal cell clusters, we identified 4 distinct cell types/states (Figure 1D). Examination of lineage-specific markers identified clusters with gene signatures of stem-like 1 (subcluster 1), multilineage progenitor-like (subcluster 2), osteoblast-like (subcluster 3), and dura/fibroblast-like (subcluster 4). Supplementary Table S1 reports the top 50 genes in each of the 4 subclusters. EGFP was widely expressed among those 4 subclusters with particularly high expression in multilineage progenitor-like cells (subcluster 2). Stem cell markers, including lymphocyte antigen 6 family member A (*Ly6a)*, *Pdgfrα*, *Cd34*, and *Thy1*, showed predominant expression in subcluster 1 (stem-like cells), with some expression in subcluster 2 as well. Multilineage progenitor markers Scleraxis (*Scx*), Nestin (*Nes*), S100 calcium binding protein A6 (*S100a6*), and Actin alpha 2 (*Acta2*) showed enriched expression in subcluster 2. Osteoblasts markers Bone gamma-carboxyglutamate protein (*Bglap*), Bone gamma-carboxyglutamate protein 2 (*Bglap2*), Collagen, type I, alpha 1 (*Cola1*) and Secreted phosphoprotein 1 (*Spp1*) displayed enriched expression in subcluster 3. Subcluster 4 was categorized as demonstrating a dura/fibroblast-like signature, including expression of Matrix gla protein (*Mgp*), C-X-C motif chemokine ligand 12 (*Cxcl12*), Forkhead box protein C1 (*Foxc1*), and Forkhead box protein D1 (*Foxd1*) (Figure 1E).

To understand the potential relationship among mesenchymal cells clusters, we used Monocle2 to order single cells and constructed an entire lineage differentiation trajectory with a tree-like structure (Figure 1F, G). Our trajectory analysis identified a continuum of cells featuring a single distinct branch point. This branch originated predominantly from the stem cell population and split into 2 divergent branches, terminating in dura/fibroblast-like cells and osteoblasts. The less differentiated branch primarily consisted of MLP cells, suggesting an intermediate transitional state. Analysis of gene expression patterns across pseudotime revealed a progressive decline in stem cell-associated genes such as *Cd34* and *Thy1* (Figure 1H). Genes associated with MLP peaked in the early stage. As pseudotime advanced, gene expression associated with osteogenesis (*Bglap*, *Spp1*) and fibrogenesis (*Foxc1*, *Mgp*) increased (Figure 1H). Trackplot further showed high EGFP expression in MLP-like cells (subcluster 2) and osteoblast-like cells (subcluster 3), while dura/fibroblast-like cells showed lower and more variable EGFP expression (subcluster 4), and stem-like cells showed the lowest EGFP reporter expression (subcluster 1) (Figure 1I). Analysis of EGFP reporter distribution was further assessed among the 4 mesenchymal subclusters. Using read count ≥ one as a threshold for positive, a high frequency of EGFP^+^ cells was seen across mesenchymal subclusters (Figure 1J). Quantification of this UMAP showed that nearly 50% of each subcluster consisted of EGFP^+^ cells (Figure 1K).

Taken together, our single-cell sequencing analysis demonstrates that Pdgfrα is prominently expressed in the mesenchymal cells within a calvarial injury site, although both the distribution and intensity of Pdgfrα reporter activity varies across mesenchymal cell subclusters.

### Healing pattern in calvarial bone defects

To investigate the involvement of Pdgfrα^+^ cells in cranial bone repair, we initiated our study by using an established calvarial bone defect model in Pdgfrα^mT/mG^ reporter mice, with multiple time points for evaluating the defect healing pattern and expression of the Pdgfrα^+^ reporter (0, 3, 7, 14, and 28 days after injury) (Figure 2A). Initially, a precise circular defect measuring 1.8 mm in diameter was created in the frontal bone. Subsequently, we tracked the process of re-ossification over a 4-week period using micro-computed tomography (μCT) scans (Figure 2B). Upon evaluating the day 0 group (representing the immediate aftermath of injury), a distinct defect featuring a smooth perimeter was evident. Notably, by day 3, sparse yet noticeable instances of healing bone were observed along the periphery of the defect sites, suggesting an initial bone healing response originating from the edges of the defect. As the timeline progressed to day 7 postinjury, all margins of the defect exhibited signs of new bone formation surrounding the defect edges. By the 2-week timepoint, substantial quantities of newly generated bone were evident at the defect margins. At the 4-week milestone postinjury, the newly formed bone had effectively covered over half of the original defect area (Figure 2B). These observed differences were subsequently substantiated through quantitative parameters, encompassing metrics such as bone volume (BV) (Figure 2C), percentage bone volume (BV/TV) (Figure 2D), the average diameter of the remaining non-ossified bone defect (Figure 2E), bone fractional area (BFA) (Figure 2F), and a semiquantitative bone healing score (Figure 2G).

### Pdgfrα reporter activity following calvarial bone injury

It is worth noting that EGFP signal was observed in the uninjured calvaria after tamoxifen administration, including the periosteum, endosteum, and dura in the frontal bone. Moreover, high Pdgfrα reporter activity was noticed especially in coronal suture and interfrontal suture (Figure 3). Within the immediate injured bone, this defect site appeared to be devoid of any discernible tissue, as revealed by H&E staining (Figure 4A; Supplementary Figure S2 for high magnification images). Pdgfrα reporter was present within the interfrontal and coronal sutures, periosteum, and dura mater (Figure 4B). On day 3 postinjury, a thin layer of fibro-inflammatory tissue had developed (Figure 4A). A significant increase in Pdgfrα reporter activity in the fibro-inflammatory defect area was noted, most notably adjacent to the injured bone (Figure 4B). At later time points on day 7, with obvious immature bone tissue and more fibro-inflammatory structures in the defect region (Figure 4A), the majority of the fibro-inflammatory tissue filled the defect area that mostly expressed PDGFRα (Figure 4B). On day 14, with less fibrous tissue occupied in the defect area (Figure 4A), the majority of EGFP reporter activity was localized to bone-lining cells along the re-growing defect edges corresponding to re-ossification (Figure 4B). At a later time point (day 28), a large number of mature bone tissue was seen in the defect area (Figure 4A). Pdgfrα reporter activity waned but was still present in bone-lining cells (Figure 4B). Semiquantitative analysis of relative Pdgfrα reporter activity showed a slight increase in Pdgfrα reporter activity at 3 days and a peak expression at day 7. Reporter activity waned on days 14 and 28 (Figure 4C). These data indicate that Pdgfrα reporter^+^ cells populated the calvarial defect site at early timepoints. Moreover, these Pdgfrα^+^ cells within the periosteum, dura, and sutures mark a major skeletal mesenchymal pool that contributed to calvarial defect healing in mice.

### Pdgfrα^+^ cell progeny participates in cell proliferation and osteogenesis

Our previous data have suggested that Pdgfrα^+^ periosteal progenitors have high osteogenic potential when derived from long bones.^29^ Here the proliferation and osteogenic potential of Pdgfrα^+^ cell in the calvarial niche was investigated. At the 7-day mark, the vast majority of GFP-expressing cells in the defect were also Ki67 positive, revealing that a high number of Pdgfrα+ cells exhibit signs of proliferation (Figure 5A). Our scRNA-Seq data analysis also showed that most Pdgfrα^+^ cells expressed *MKi67* (Supplementary Figure S3A and B). Demonstrating the significance of *Runx2* as a pivotal transcription factor in osteogenesis, immunofluorescent staining and single-cell analysis highlights its robust expression within the Pdgfrα^+^ cell population (Figure 5B; Supplementary Figure S3C). This substantiates the notion that the Pdgfrα^+^ cells are engaged in the osteogenic differentiation process. Notably, *Col1a1* serves as an early marker of osteoblast differentiation and stands as a primary product synthesized by osteoblasts during bone formation. According to our single-cell analysis, *Col1a1* was ubiquitously expressed among the whole mesenchymal cells (Supplementary Figure S3D). The co-expression of widespread collagen type 1, as depicted in Figure 5C, alongside the GFP reporter at the site of the defect, underscores the differentiation of Pdgfrα^+^ cells toward an osteogenic pathway. These observations collectively provide compelling evidence that a large portion of Pdgfrα^+^ cells within the calvaria are not only engaged in proliferation but also actively contributing to bone formation postinjury.

### Depletion of Pdgfrα reporter^+^ progenitor cells impair calvarial repair

**Figure 6.**
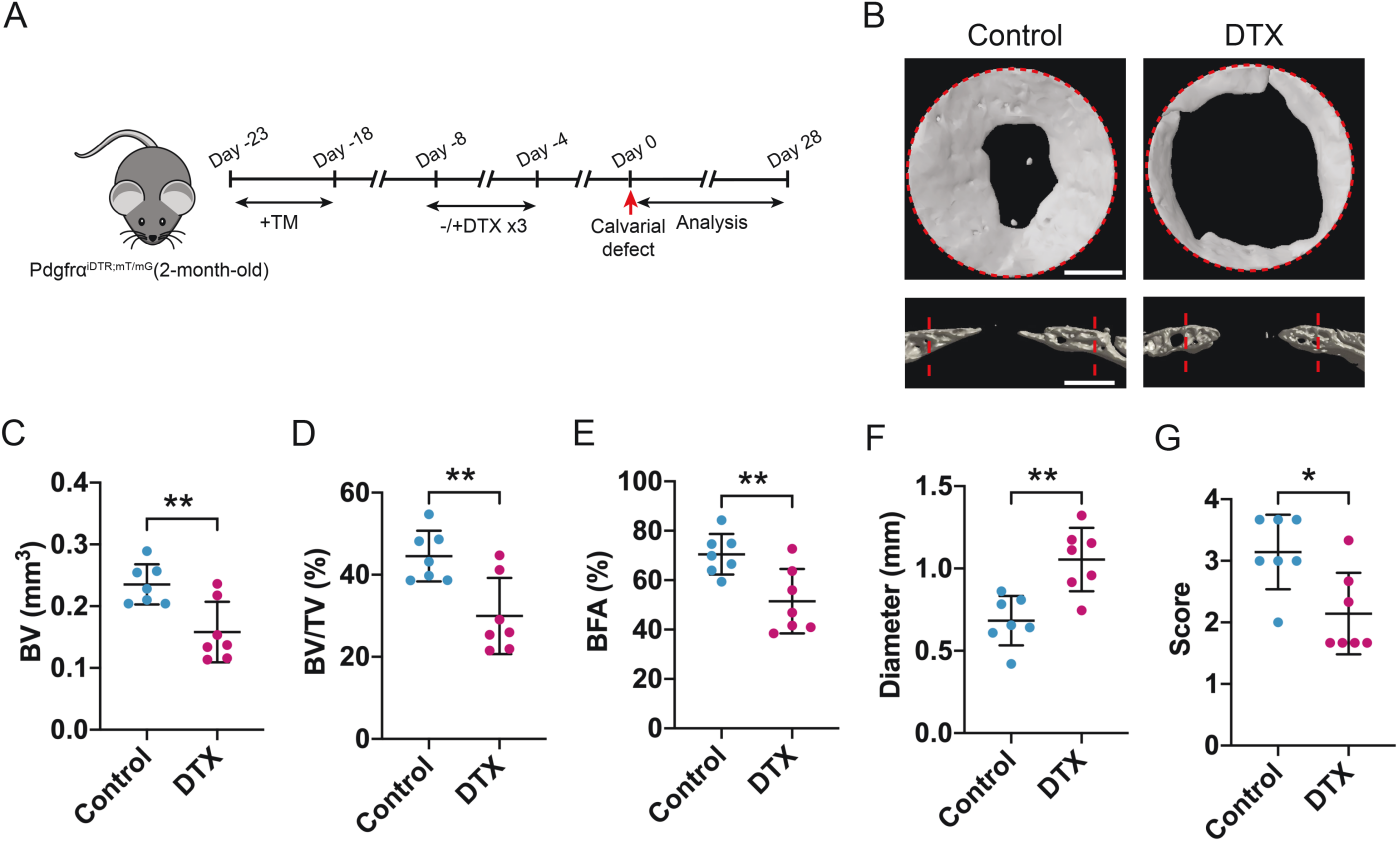
Depletion of Pdgfrα reporter + progenitor cells impairs calvarial defect healing. (A) Pdgfrα^iDTR;mT/mG^ animals (male, aged 2 months) were sequentially administered tamoxifen (TM) and diphtheria toxin (DTX), and calvarial defect was then created. The analysis was performed 28 days after injury. (B) Representative micro-CT 3D reconstructions (top view) and axial cross-sectional images (bottom) obtained 28 days after injury. The original defect margins were highlighted by dashed lines. Quantitative analysis of micro-CT images obtained at 28 days postinjury. The analysis included the (C) bone volume (BV), (D) bone volume/tissue volume (BV/TV), (E) bone formation area (BFA), (F) residual defect diameter, and (G) bone healing score (score). Each dot represents an individual mouse, and the data are represented as mean ± SD; *n* = 7 mice per group. Scale bars, 500 μm. Statistical significance was assessed by one-way ANOVA test; *P < .05; **P < .01.

To further investigate the role of Pdgfrα reporter^+^ mesenchymal cells in calvarial defect healing, Pdgfrα reporter^+^ cells were genetically ablated by crossing Pdgfrα^mT/mG^ mice with iDTR mice to generate Pdgfrα^iDTR;mT/mG^ animals. DTX-mediated ablation was then performed prior to a calvarial defect model in Pdgfrα^iDTR;mT/mG^ mice (Figure 6A). Healing among DTX and control-treated bone defects was examined over a 4-week period. Results showed a significant reduction in calvarial defect re-ossification among DTX-treated frontal bones, as visualized using a top-down view of μCT reconstruction as well as coronal cross-sectional images (Figure 6B). μCT quantification was performed to assess bone healing using multiple metrics (Figure 6C-G). The amount of regenerated bone within the defect site was quantified as bone volume (BV) (Figure 6C; 35.8% reduction), fractional BV/tissue volume (TV) (Figure 6D; 35.8% reduction), and bone fractional area (BFA) (Figure 6E; 28.5% reduction). Conversely, the mean diameter of the remaining non-ossified bone defect was significantly increased among DTX-treated samples (Figure 6F; 53.3% increase in comparison with control). In agreement with these findings, a semi-quantitative bone healing score showed a significant reduction among DTX-treated bone defect (Figure 6G, 32.5% reduction).

**Figure 7.**
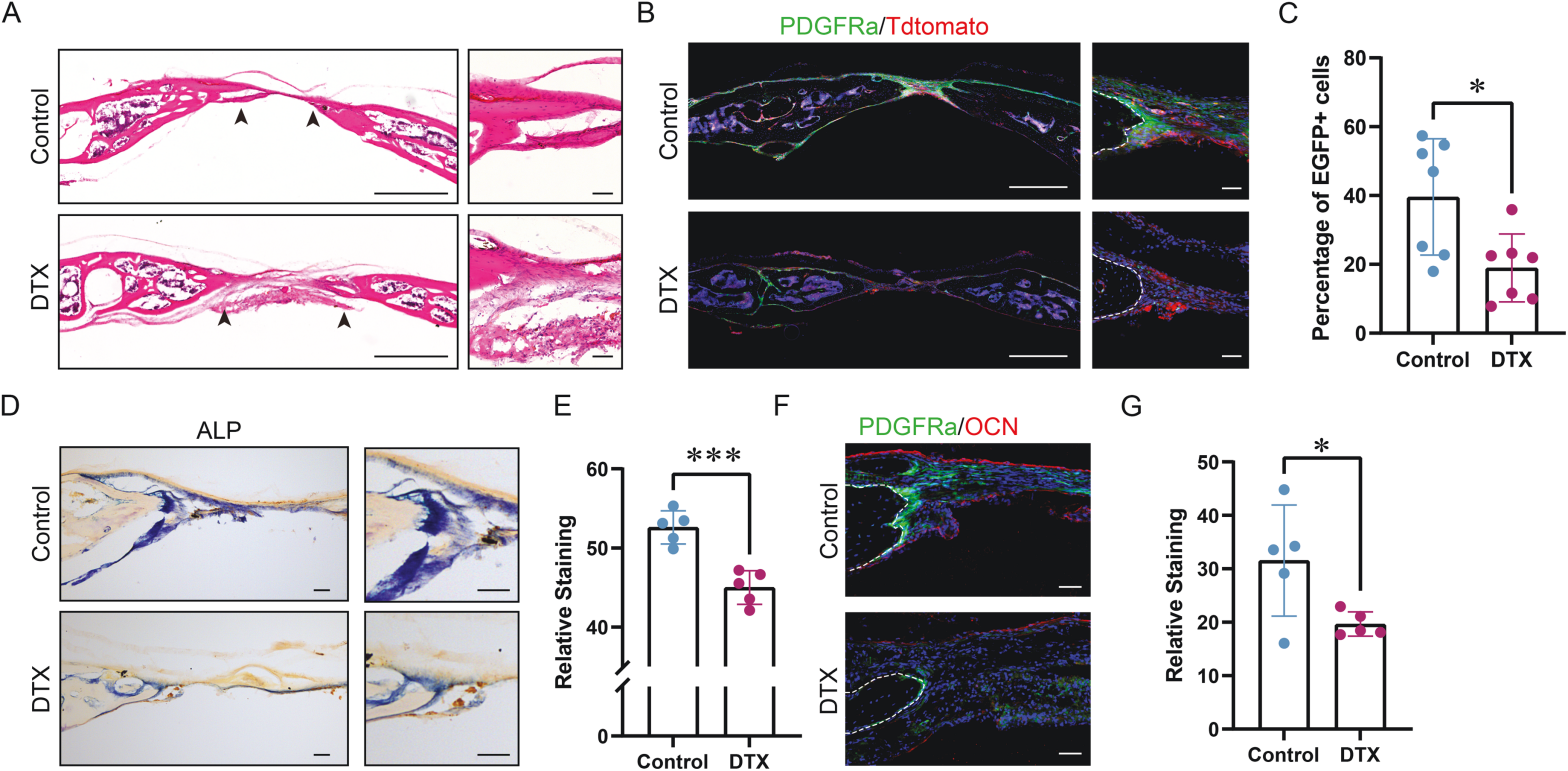
Histology of calvarial defect healing following depletion of Pdgfrα reporter + progenitor cells. (A) Representative H&E-stained images of calvarial defect in Pdgfrα^iDTR;mT/mG^ animals, 28 days postinjury. Black arrowheads indicate healing bone edges. (B) Representative tile scans and high-magnification images of calvarial defects in Pdgfrα^iDTR;mT/mG^ reporter animals, 28 days postinjury. (C) Evaluation of Pdgfrα reporter + activity as the percentage of positive cell numbers within the defect area 28 days after injury (*n* = 7 mice). (D) Representative image of alkaline phosphatase (ALP) staining of the bone defect edge. (E) Quantitative analysis of ALP staining. The relative ALP staining density was represented using mean integrated pixel density (*n* = 5 mice). (F) Representative image of osteocalcin (OCN) immunohistochemical staining in Pdgfrα^iDTR;mT/mG^ injured calvarial defect animals. (G) Quantification of OCN staining in term of relative fluorescence (*n* = 5 mice). Scale bar: 100 μm. Each dot represents a single animal. Data are represented as mean ± 1 SD. *P < .05, **P < .01, and ***P < .001 as assessed by two-tailed Student’s *t* tests.

Next, H&E staining confirmed a significant reduction in calvarial re-ossification, including a widened distance between bony fronts in DTX-treated group. (Figure 7A; black arrowheads indicate defect edge). Cell depletion efficiency was confirmed via immunofluorescent detection of reporter activity, reflecting a 52% reduction in Pdgfrα reporter activity (Figure 7B and C). A significant reduction of alkaline phosphatase (ALP) activity (13.6% reduction) was observed in DTX-treated animals by ALP staining and quantification (Figure 7D and E). OCN immunohistochemical staining was next performed and confirmed a significant reduction in OCN^+^ osteoblastic numbers with DTX treatment animals (Figure 7F), reflecting a 37.7% reduction in OCN immunostaining (Figure 7G).

## Discussion

In this work, by combining single-cell RNA sequencing analysis, inducible lineage tracing reporter mice and cell ablation strategies, we confirm that Pdgfrα^+^ cells, which are identified as a subset of mesenchymal stem cells within the calvarial niche, are highly responsive to calvarial injuries. These PDGFRα-expressing cells are essential for the proper repair of calvarial bone.

The relationship between Pdgfrα^+^ cells and other skeletal progenitor cell markers has been extensively reported, indicating a notable overlap among these markers. FACS analysis of periosteal cells from femurs, tibias, pelvis, and calvaria of Prx1-CreER-GFP mice displayed that over 80% of Prx1^+^ periosteal cells exhibit high cell surface expression of CD140a (Pdgfrα).^41^ Furthermore, it was observed that a significant majority of LepR^+^ periosteal cells, approximately 82%, also express CD140a.^41^ Shi et al. reported that approximately 80% of Gli1^+^ cells located under the growth plate of long bones express Pdgfrα, and more than 60% of cells from the chondro-osseous junction of long bones are Pdgfrα positive.^5^ It has also been shown that Prx1 reporter^+^ long bone periosteal stem cells express higher levels of Pdgfrα compared to the Prx1-negative population.^9^ Sivaraj et al., utilizing a genetic reporter for OSX expression in conjunction with Pdgfrα-GFP, confirmed the presence of cells positive for both markers in trabecular and cortical bone, as well as in the epiphysis of the femur.^42^ Within the calvarial niche, an overexpression of Pdgfrα also observed in Prx1^+^ cells isolated from calvarial sutures of 4-week-old Prx1-CreER-EGFP^+^ mice compared with EGFP^−^ cells.^8^ Ortinau et al. demonstrated Mx1^+^αSMA^+^ cells maintained a high expression of CD140a in calvarial injury sites.^41^ These data in combination with our own suggest that high Pdgfrα expression is a common feature of mesenchymal-like cells within the skeleton, although it most likely must be used in combination with other markers.

The skeleton has different requirements for the functionality of tissue-resident progenitors at different life stages. The functional significance of Pdgfrα in development is evident. Only a small percentage of the Pdgfrα-null mice can survive past birth.^43^ He et al. explored PDGFRα expression and the effects of its dysregulated activity on calvarial development before E18.5, revealing PDGFRα’s expression in the coronal suture, as well as in subsets of endosteal and periosteal osteoprogenitors, demonstrating its critical role in normal calvarial development.^44^ In a subsequent study, they found that Pdgfrα is vital for calvarial morphogenesis, affecting both the frontal and parietal bones due to Pdgfrα deficiency.^32^ Moreover, they identified that Pdgfrα as a novel player of chondrocranial cartilage development.^45^ The role of Pdgfrα in the adult pathophysiological skeletal context has not yet been completely understood. Pdgfrα expression in the periosteum and endosteal which attributed to fracture repair have been proved by several studies.^16,29,46^ In the present study, Pdgfrα^+^ cells can be observed in periosteum, suture, and dura of uninjured calvaria which consist with the observance of its expression during calvarial development.^32^ Bone repair can proceed by either endochondral ossification or intramembranous ossification. Our previous study suggested that Pdgfrα cells within the periosteum act as a stem cell reservoir for periosteal fracture repair. It is generally believed that calvaria defect repair only through intramembranous ossification. By utilizing an inducible Pdgfrα reporter animal model, we confirmed that Pdgfrα^+^ cells contribute to the healing process of calvarial defects, with notable expression on day 7 postinjury, and that Pdgfrα activity is closely associated with cell proliferation and osteogenic differentiation. Ablation of Pdgfrα^+^ cell numbers can significantly result in an impaired calvarial bone repair. Integrating our findings from both long bone fracture repair and calvarial studies, we propose that Pdgfrα^+^ progenitor cells play a role in both endochondral and intramembranous ossification processes.

There are several limitations to the present study. Firstly, the ablation of Pdgfrα reporter^+^ cells mediated by DTX is not exclusive to bone tissue, thereby potentially affecting a broad range of tissues beyond the skeletal system. We cannot rule out the possibility that depleting Pdgfrα reporter + cells from other tissues might indirectly impact the functionality of the periosteum, sutures, or dura. In addition, we acknowledge that the depletion efficiency in our system is only partial as shown in the current and previous work by our laboratory.^29^ Enhanced depletion efficiency of Pdgfrα+ cells may demonstrate an even more robust phenotype. Moreover, while analysis of defect tissues at 4 weeks postinjury is a standard timepoint for calvarial defect healing in the 1.8 mm frontal bone defect model,^35,36,47,48^ using a single timepoint to evaluate depletion efficiency is a limitation in our study. Therefore, future assessments at later timepoints would provide valuable insights. Furthermore, the primary source of Pdgfrα^+^ cells in the area of the defect—whether from the periosteum, sutures, or dura—remains uncertain. A previous study has showed that Gli1^+^ cells in the suture mesenchyme contribute to injury repair by using lineage tracing analysis and Gli1 inducible reporter system.^6^ In the present study, dense PDGFRα reporter activity could be observed in the interfrontal and coronal suture of uninjured calvarial, indicating that suture residing Pdgfrα^+^ cells might be the main source contributing to the calvarial defect healing.

## Conclusion

In summary, we have identified the presence of Pdgfrα^+^ mesenchymal progenitor cells in the calvarial injury niche. The deletion of these Pdgfrα^+^ cells resulted in impaired calvarial bone healing postinjury, highlighting the significant role of Pdgfrα^+^ cells in maintaining bone homeostasis and facilitating repair within calvarial bone.

## Supplementary material

Supplementary material is available at *Stem Cells Translational Medicine* online.

szae041_suppl_Supplementary_Figures_S1-S3_Tables_S1

## Data Availability

The data underlying this article will be shared on reasonable request to the corresponding author.
