# Supplementary material for: Requirement of Pdgfrα+ cells for calvarial bone repair
Source: Stem Cells Transl Med. 2024 Jul 10;13(8):791–802. doi: 10.1093/stcltm/szae041 (PMC11328938; doi:10.1093/stcltm/szae041)
Supplement: szae041_suppl_Supplementary_Figures_S1-S3_Tables_S1 [file szae041_suppl_supplementary_figures_s1-s3_tables_s1.docx]

**Requirement of Pdgfrα^+^ cells for calvarial bone repair**

Xin Xing^1#^, Zhao Li^1#^, Jiajia Xu^1#^, Austin Z Chen^1^, Mary Archer^1^, Yiyun Wang^1^, Mingxin Xu^1^, Ziyi Wang^1^, Manyu Zhu^1^, Qizhi Qin^1^, Neelima Thottappillil^1^, Myles Zhou^1^, Aaron W James^1*^.

^1^Department of Pathology, Johns Hopkins University, Baltimore, MD 21205, USA

^#^These authors contributed equally to this work.

* **Correspondence:** Aaron W. James, M.D., Ph.D., Department of Pathology, Johns Hopkins University, Ross Research Building, Room 524A, 720 Rutland Avenue, Baltimore MD 21205; Tel: (410) 502-4143; Fax: (410) 955-0394; Email: [awjames@jhmi.edu](mailto:awjames@jhmi.edu)

**Supplementary Tables and Figures**

**Supplementary Table S1. Top 50 enriched genes in each four subclusters of mesenchymal cells within Pdgfrα^mT/mG^ mice.**

| **Subcluster 1: Stem** | **Subcluster 2: MLP** | **Subcluster 3: Osteo** | **Subcluster 4: Dura/fibro** |
| --- | --- | --- | --- |
| Dnm1 | Vim | Bglap2 | Mgp |
| Ssc5d | Actb | Bglap | Apoe |
| Cd276 | Actg1 | Col1a1 | Ecrg4 |
| P4ha3 | Lgals1 | Col1a2 | Slc38a2 |
| Fam102b | Rpl13 | Col11a2 | Cst3 |
| Olfml2b | Rps3 | Bglap3 | Fth1 |
| Epb41l3 | Postn | Cfh | Foxd1 |
| Mcub | Rack1 | Sparc | Itm2b |
| Itgb3 | Rpl8 | Spp1 | Smoc1 |
| Gm47283 | Ppia | Cadm1 | Fxyd5 |
| Arhgap42 | Rpl12 | Col22a1 | Foxp2 |
| Axl | Tmsb4x | Fat3 | Igf2 |
| Sh3gl1 | Col3a1 | Satb2 | Shisa3 |
| Pxdc1 | Rpsa | Serpinf1 | Mdk |
| Pcdh19 | Col6a3 | Car3 | Plpp3 |
| Gpr153 | Myl6 | Col11a1 | Eya2 |
| Dbn1 | Rpl18 | Ano1 | Slc4a10 |
| Abhd2 | Hsp90ab1 | Mlip | Lmo4 |
| Phldb1 | Rps5 | Pdzd2 | Emb |
| Ece1 | Rpl15 | Smpd3 | Crispld1 |
| Sh3kbp1 | Nme2 | Cpe | Rbp1 |
| Pde8a | Col5a2 | Ifitm5 | Gdf10 |
| Zfp469 | Txn1 | Col13a1 | Gnas |
| Aida | Col5a1 | Slc8a3 | Hmgcs2 |
| Slc41a2 | Rps6 | Fign | mt-Nd2 |
| Elf4 | Rps7 | Frmd4b | mt-Cytb |
| Mvp | Ybx1 | Mef2c | S100b |
| Il3ra | Rpl13a | Slc36a2 | Cdkn1c |
| Srpx2 | Rpl10a | Rerg | Ctnnd2 |
| Snap23 | Anxa2 | Omd | Tmtc1 |
| Pdia5 | Calm1 | Kcnma1 | Ppm1h |
| Chst12 | Cthrc1 | Sgms2 | Cpz |
| Pip5k1a | Col6a1 | Dapk2 | Rspo3 |
| Sept8 | Serpinh1 | Tent5a | Cdh11 |
| Ugcg | Acta2 | Lipc | Slc23a2 |
| Rbpj | Eef1b2 | Cdh2 | Zbtb20 |
| Itpripl2 | Rpl7 | Sorbs2 | Rassf2 |
| Agfg1 | Rps16 | Insc | Fgl2 |
| Gramd3 | Rpl5 | Lmo7 | Kctd12 |
| Ugp2 | Rpl14 | Ibsp | Ism1 |
| Mfge8 | Ptma | Fhod3 | Cpxm1 |
| Tbc1d2b | S100a6 | Ptgis | Glul |
| Mfhas1 | Anxa5 | Cgref1 | Lbp |
| Hsd17b12 | Rpl29 | Ppfia2 | Tspan13 |
| Marveld1 | Timp1 | Robo2 | Adamts9 |
| Gas2l1 | Rps20 | Dtna | Foxc1 |
| Lamb1 | Tuba1a | Nupr1 | Smpdl3a |
| Lamc1 | Cfl1 | Sp7 | Atp1b1 |
| Map3k20 | S100a9 | Vdr | mt-Nd4 |
| Fyn | Cd63 | Myo1d | Foxp1 |

**Osteo, osteoblast; MLP, multilineage progenitor; Dura/fibro, dura/fibroblast.**


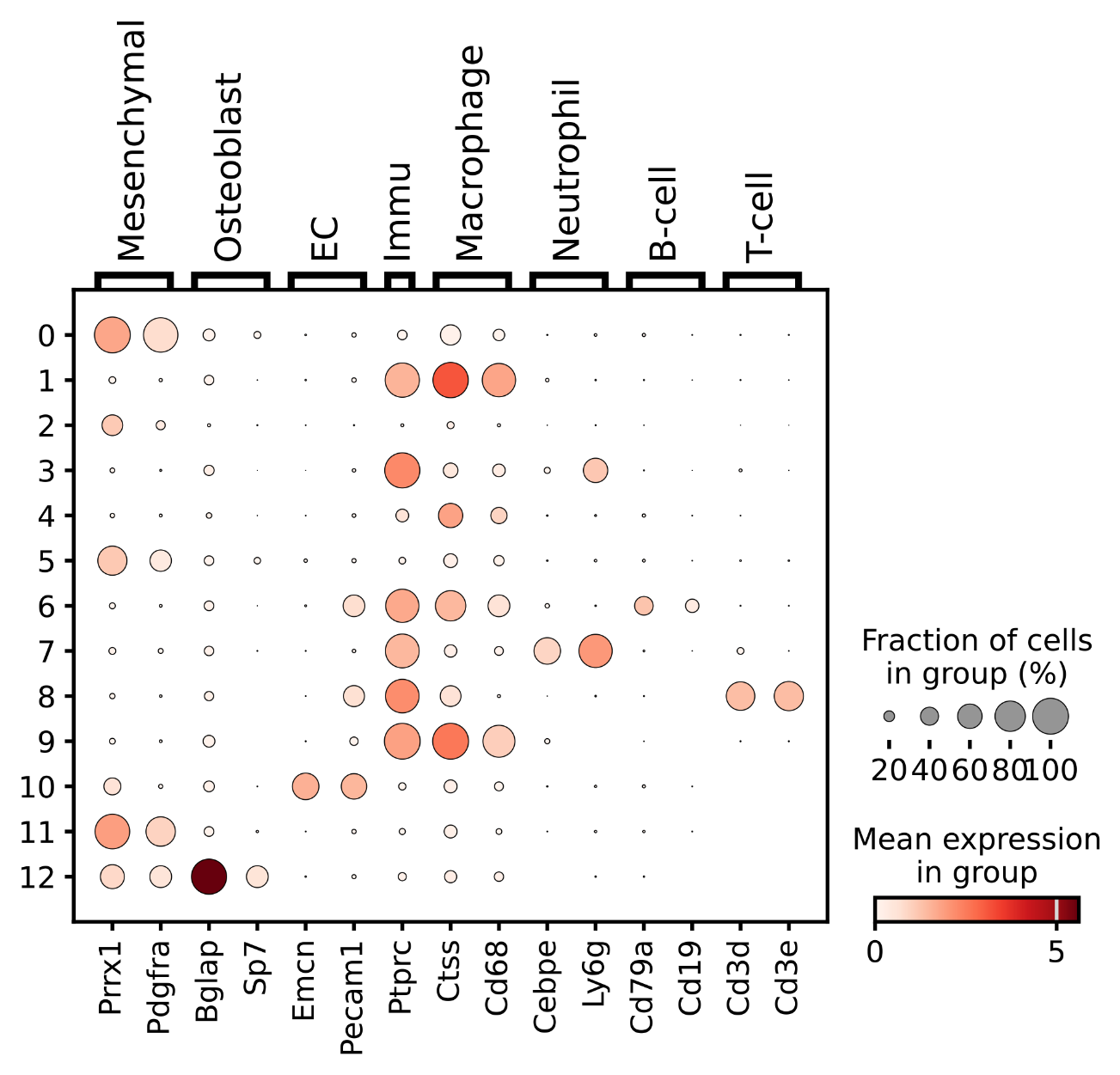


**Supplementary Figure S1.** Dot plot displaying expression of cluster defining genes. EC, endothelial cell; Immu, Immune Cells. Dot size reflects the percentage of cells in a cluster expressing each gene; dot color reflects expression level.


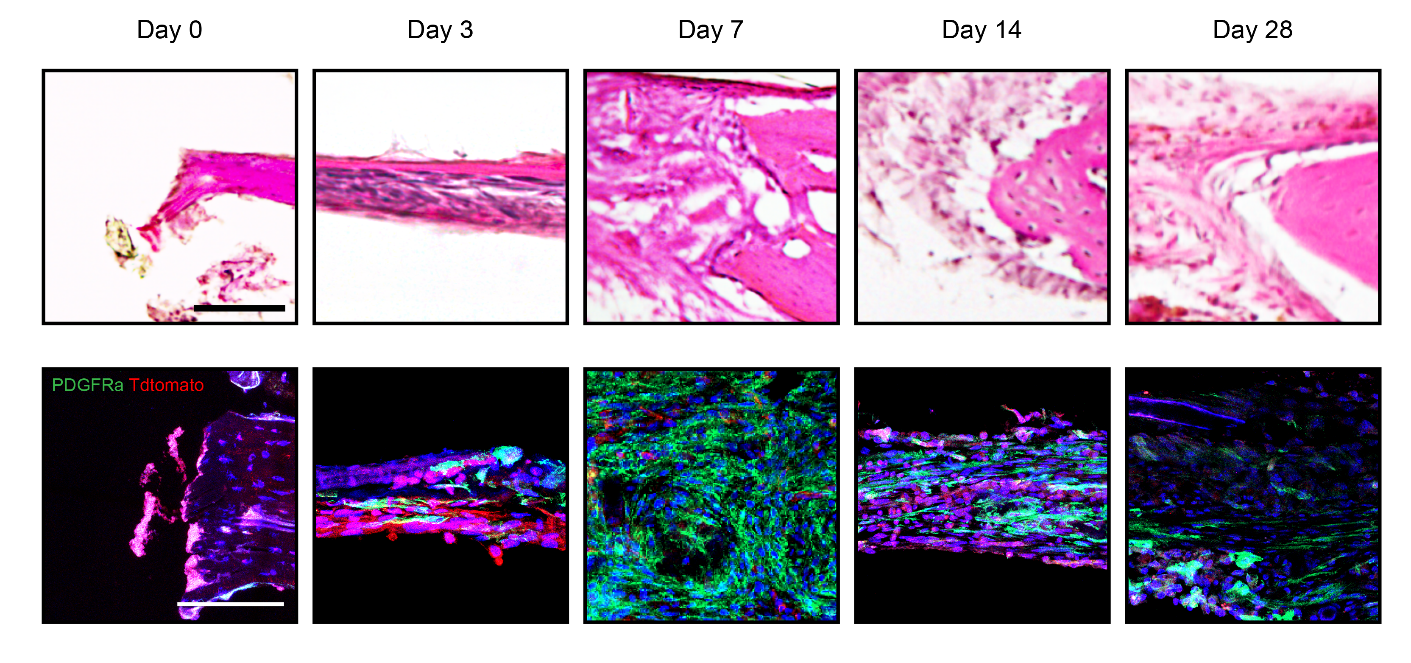


**Supplementary Figure S2.** Representative high magnification images of haematoxylin and eosin (H&E) staining (upper panel), and reporter activity (lower panel) of coronal cross sections of the healing defect site at different time points after injury (0, 3, 7, 14, and 28 d). Scale bar: 100μm.


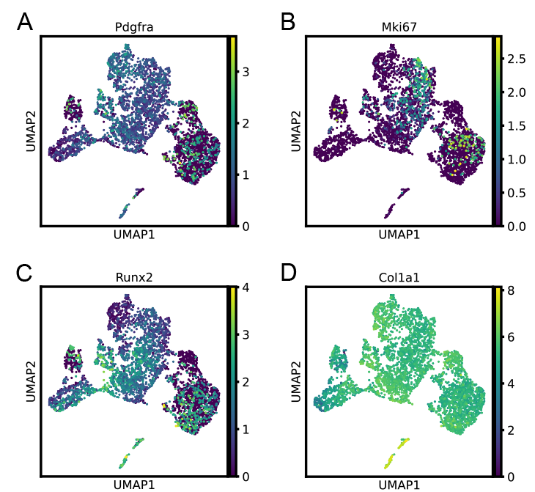


**Supplementary Figure S3.** Feature plot showing the expression pattern of (A) *Pdgfra*, (B) *Mki67*, (C) *Runx2*, and (D) *Col1a1* in the mesenchymal subpopulations from the calvarial defect site of Pdgfrα^mT/mG^ mice by single-cell analysis.
